# Expansion of Double-Negative T Cells in Patients before Liver Transplantation Correlates with Post-Transplant Infections

**DOI:** 10.3390/jcm11123502

**Published:** 2022-06-17

**Authors:** Hong Lei, Min Tian, Xiaogang Zhang, Xuemin Liu, Bo Wang, Rongqian Wu, Yi Lv

**Affiliations:** 1 Key Laboratory of Precision Medicine to Pediatric Diseases of Shaanxi Province, Shaanxi Institute for Pediatric Diseases, The Affiliated Children’s Hospital of Xi’an Jiaotong University, Xi’an 710003, China; hong.lei@xjtu.edu.cn; 2 National Local Joint Engineering Research Center for Precision Surgery and Regenerative Medicine, The First Affiliated Hospital of Xi’an Jiaotong University, Xi’an 710061, China; rwu001@mail.xjtu.edu.cn; 3 Department of Hepatobiliary Surgery, The First Affiliated Hospital of Xi’an Jiaotong University, Xi’an 710061, China; tianmin721@163.com (M.T.); little_gang@163.com (X.Z.); a1090224@163.com (X.L.); bobwang75@sina.com (B.W.)

**Keywords:** double negative T cells, T cell exhaustion, liver transplantation, infections, immunosuppressive therapy

## Abstract

Liver transplantation (LTx) is currently the only effective therapy for patients with end-stage liver diseases, but post-transplant infection is a key issue for morbidity and mortality. In this study, we found that pre-transplant patients with an expansion of double-negative T (DNT) cells (CD3^+^CD4^−^CD8^−^ T cells) had an increased incidence of infections within the first 6 months after LTx. These DNT cells also negatively correlated with their CD4/CD8 ratio. Compared to patients who had no infections after LTx, these DNT cells expressed more CD25, especially in the memory compartment. The receiver operating characteristic (ROC) analysis showed that the threshold area under the ROC curve of DNT cells which could be used to distinguish LTx patients with post-transplant infections from patients without infections after LTx was 0.8353 (95% CI: 0.6591–1.000). The cut-off for the pre-LTx DNT cell level was 11.35%. Although patients with post-transplant infections had decreased levels of CD4/CD8 T cells, CD8^+^ T cells in these patients were more exhausted, with higher PD-1 expression and lower IFNγ secretion. The increased levels of DNT cells in patients with post-transplant infections were still observed 2 weeks after LTx, with higher proportions of memory DNT cells. In conclusion, increased levels of DNT cells in pre-LTx patients may be valuable for the prognosis of post-transplant infections, especially within the first 6 months after LTx.

## 1. Introduction

Liver transplantation (LTx) is currently the most powerful treatment for patients with end-stage liver disease. With the development of both surgical techniques and immunosuppressants, excellent 1-year survival rates of >89% have been achieved; however, infections are still one of the key issues for morbidity and mortality in transplant patients [1,2]. Life-long immunosuppressive therapy has inhibited T cell-mediated alloimmunity, thus leading to a better allograft acceptance. However, the generally compromised T cell immunity in liver transplant recipients also increased the susceptibility to infections in these patients, especially within the first 90 days after LTx [3,4,5,6,7]. Therefore, developing predictive biomarkers for infections after LTx is essential for the optimized care and precise immunotherapy of LTx patients [8]. Many studies concerning this, as pertaining to solid organ transplantation, have been performed. Pre-transplant levels of Human Leukocyte antigen-G molecules proved useful for the identification of heart recipients who were at risk of serious infections [9]. Higher serum soluble CD30 levels were associated with an increased risk of bacterial infection after kidney transplantation [10]. Concentration of chemokines such as CXCL10 in plasma from whole blood stimulated with the specific Leishmnia antigen was found to be a new biomarker of asymptomatic Leishmania infantum infection in solid-organ transplant recipients [11]. In LTx recipients, T cell repertoire clonality was a novel biomarker predictor for sepsis, before the development of clinical symptoms [12]. However, TCR repertoire clonality analysis, using second-generation sequencing, is quite expensive and time-consuming. More biomarkers predicting post-transplant infections are still needed. Mysore et al. have reported that patients with the highest expression of PD-1^+^Tim-3^+^ T cells in the memory compartment before LTx had increased incidence of infections after LTx, especially within the first 90 days [13]. The evaluation of T cell subsets has played an important role in predicting post-transplant infections and allograft rejections in transplant recipients [14,15,16,17].

Double-negative (DN) T cells are a unique type of regulatory T cell, but the origin of these cells is controversial. They are typically defined by T cell receptor (TCR) αβ^+^CD3^+^CD4^−^CD8^−^ cells in human [18]. DNT cells only account for 3% to 5% of total T cells in the peripheral blood of healthy individuals. So far, most of the data supports the idea that DNT cells are essential for maintaining immune homoeostasis in antigen-specific ways [19,20,21]. DNT cells could express perforin, granzyme B and FasL to suppress CD4^+^, CD8^+^ T cells’, B cells’, dendritic cells’ and NK cells’ activity [22], thus protecting the allograft against rejection in organ transplantation [6,23,24,25,26,27,28]. Strategies using adoptive transfer of DNT cells also prevented the development of graft versus host disease (GVHD) and autoimmune diseases [29,30,31]. DNT cells are also an important T cell population in the liver and kidneys. In the steady state, DNT cells express high levels of CD69, CD28, CD40L and secrete IL-27, IL-10, i.a. [32]. Within 24 h after kidney ischemia-reperfusion injury (IRI), kidney DNT cells expanded significantly and suppressed the proliferation of activated CD4^+^ T cells [32,33,34]. 

Based on previous studies, we hypothesized that DNT cells may also contribute to the suppressed immunity of LTx recipients, which would therefore correlate with post-transplant infections. In this pilot study, we aimed to investigate the correlation between DNT cells in LTx recipients and post-transplant infections. We have included patient samples before and after LTx. We showed that patients before LTx with higher proportions of DNT cells had increased incidence of infections within 6 months after LTx. Furthermore, these DNT cells in patients with post-transplant infections were more activated and may correlate with T cell exhaustion in these patients. This work is valuable for identifying more biomarkers for the prediction of post-transplant infections and the optimized management of patients after LTx.

## 2. Materials and Methods

After informed consent, peripheral blood samples for research in this study were taken from patients, within 24 h before LTx and 2 weeks after LTx. Patients with post-transplant infections were defined as patients who had signs of infection and positive microbiologic cultures or viral polymerase chain reactions (PCRs) from blood samples that required altered treatment. Patients with acute rejections and early allograft dysfunction within 6 months after LTx were excluded from the final data analysis. Blood samples from age- and gender-matched, healthy volunteers were obtained from Xi’an, China. The study was approved by the First Affiliated Hospital of Xi’an Jiaotong University in Xi’an, China (Institutional Review Board, No.: 2019 G-213). All participants signed a written informed consent form to allow the collection of peripheral blood samples for research purposes only.

Whole blood was diluted with PBS and transferred onto Ficoll for gradient centrifugation within 6 h after withdrawal. Peripheral blood mononuclear cells (PBMCs) were isolated and re-suspended in RPMI 1640 media with 10% FBS for T cells’ flow cytometric phenotyping and functional experiments. The flow cytometric staining protocol was similar to that which we previously described [35]. The following antibodies were used for the surface staining: CD3 (SK7), CD4 (A161A1), CD8 (HIT8a), CD25 (M-A251), CD127 (eBioRDR5), CD45RA (HI100), CD62L (DREG-56), PD-1 (EH12.2H7), and Tim-3 (F38-2E2) from Biolegend. LIVE/DEAD1 Fixable Aqua Dead (Invitrogen, Paisley, UK) was used to exclude dead cells. FoxP3 (259D/C7) from BD bioscience was stained intracellularly with the FoxP3 staining buffer and protocol (eBiosciences, San Diego, CA, USA). Data were acquired by a NovoCyte Flow Cytometer (Acea Biosciences Inc., Agilent, MA, USA). The analysis was performed with Flowjo Software 9.2 (TreeStar, Ashland, OR, USA). The gating strategy of the T cell subsets were the same as we described in a previous study [27]. Briefly, lymphocytes were gated based on FSC and SSC, then doublets were excluded using FSC and SSC height versus area characteristics, and further dead cells were excluded using LIVE/DEAD Fixable Aqua Dead. Then, T cells subsets were gated using CD3, CD4, CD8 and other markers, based on unstained controls. 

Freshly isolated PBMCs were stimulated with a cell activation cocktail (PMA and ionomycin) from Biolegend, in the presence of brefeldin A for 5 h. Then, CD3 (SK7) and CD8 (HIT8a) were used for surface staining, and LIVE/DEAD1 Fixable Aqua Dead (Invitrogen, Paisley, UK) was used to exclude dead cells. TNFα (MQ1-17H12), IFNγ (4S.B3), Interleukin 2 (MQ1-17H12), and IL17 (BL168) were stained intracellularly with the FoxP3 staining buffer (eBiosciences, San Diego, CA, USA). These antibodies were purchased from Biolegend. Data were acquired on the NovoCyte Flow Cytometer (Acea Biosciences Inc., Agilent, MA, USA). Statistical analysis was conducted using GraphPad Prism 8 (GraphPad Software, La Jolla, CA, USA). Significance was defined as *p* < 0.05 (* *p* < 0.05; ** *p* < 0.01; *** *p* < 0.001). Comparisons between the two groups were performed by either an unpaired *t*-test or the Mann–Whitney test. The receiver operating characteristic (ROC) analysis of DNT cells and patients with post-transplant was performed using GraphPad Prism 8 (GraphPad Software, La Jolla, CA, USA).

## 3. Results

### 3.1. Subsection

#### 3.1.1. Characteristics of LTx Patients

This study is based on LTx patients who underwent orthotopic LTx at the First Affiliated Hospital of Xi’an Jiaotong University, China. A total of 19 patients who met our study criteria, with complete clinical information and 6 months’ follow-up visits at various time points, from the initial 30 patients were enrolled, as well as 12 age- and gender-matched, healthy controls (HCs). Patients were grouped into two groups: transplant recipients who developed infections (INF group) within 6 months after LTx and those who had no infections within the same period after LTx (NI group). The characteristics of the patients are listed in Table 1. 

There were no significant differences between the median values of age, gender, and Mayo End-Stage Liver Disease (MELD) scores between the two patient groups (Table 1). The immunosuppression regimen we used for patients without hepatocellular carcinoma (HCC) was a combination of prednisone, a calcineurin inhibitor such as CyclosporineA (CsA), and mycophenolate mofetil (MMF). Prednisone was used for the first month post-LTx and MMF was used for the first 3 years after LTx, while the calcineurin inhibitor was used long-term. Similar immunosuppression regimens without prednisone were applied for HCC patients. The concentration of the calcineurin inhibitor was monitored and the doses were adjusted to main adequate concentrations. The calcineurin inhibitors used in this study were, coincidentally, all CsA. The median concentration of CsA post-dose levels (C-2) in the two groups over 6 months are shown in Table 1, and no statistical difference was found. Regarding the primary diseases before LTx, around 1/3 patients in both groups presented with hepatocellular carcinoma (HCC); other patients presented with HBV or HCV. The HBV DNA levels of patients before LTx were less than 100 IU/mL, while the HCV RNA levels of patients before LTx were less than 1000. We followed the patients’ infectious episodes for the first 6 months after LTx; nine patients developed bacterial infections, or bacterial infections in combination with fungal infections, at least once (Table 2).

#### 3.1.2. Higher CD4/CD8 Ratio in Transplant Recipients with Infections after LTx

**Figure 1:**
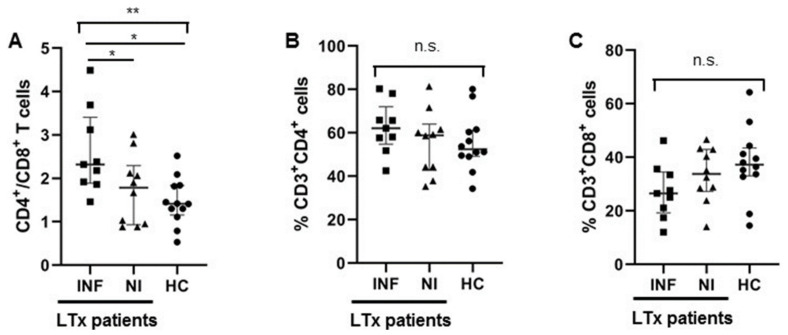
Increased ratios of CD4^+^/CD8^+^ T cells were observed in LTx patients with post-transplant infections. (**A**) Ratio of CD4^+^/CD8^+^ T cells in pre-LTx patients with post-transplant infections (INF), patients without infections after LTx (NI), and healthy controls (HC) were shown individually. (**B**,**C**) The proportion of CD4^+^ T cells and CD8^+^ T cells in the two groups of patients (INF & NI) and the healthy controls were shown individually. Comparison between the three groups was undertaking through one-way ANOVA, followed by Tukey’s multiple comparison test. (INF: n = 9; NI: n = 10; HC: n = 12). * *p* < 0.05, ** *p* < 0.01.

We investigated the T cell heterogeneity and function in patients before LTx using multicolor flow cytometry. Firstly, the CD4/CD8 ratio was higher in the INF group than the NI group (Figure 1A). However, if we compared proportion of CD4^+^ and CD8^+^ T cells separately, no significant differences were found (Figure 1B,C). Therefore, the CD4/CD8 ratio was more important than the proportion of each T cell population.

#### 3.1.3. More DNT Cells Were Observed in Pre-LTx Patients Who Developed Post-LTx Infections

**Figure 2:**
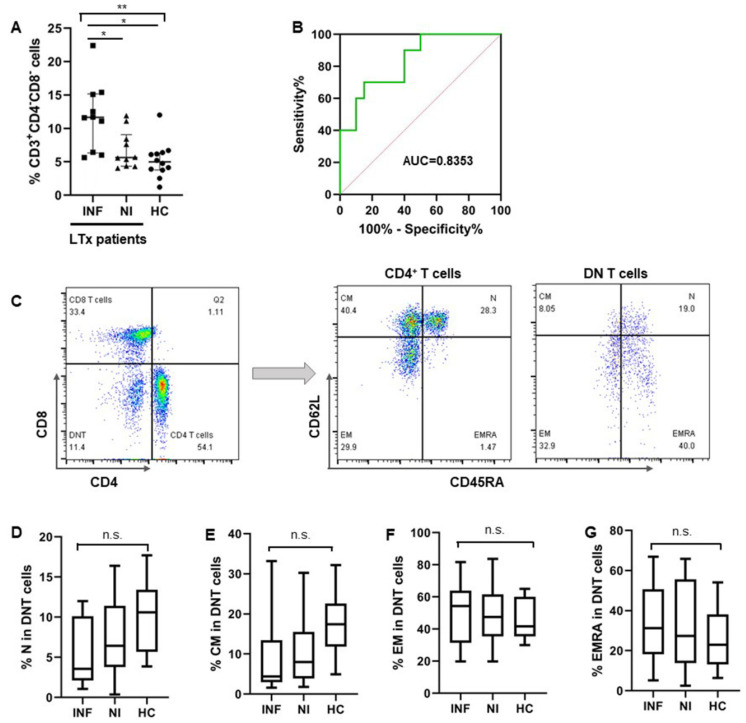
Increased DNT cells in pre-LTx patients correlated with infections after LTx. (**A**) The proportion of DNT (CD3^+^CD4^−^CD8^−^) cells in the two groups of patients (INF & NI) and healthy controls were shown separately. (**B**) The receiver operating characteristic (ROC) curve of circulating DNT cells was shown to distinguish patients who developed infections from those without infections after LTx. (**C**) Flow cytometry gating strategy of DNT cells and T cells subsets including naïve, CM, EM, and EMRA from a representative patient. (**D**–**G**) Proportion of each cell subset in 2 patients’ groups (INF & NI) and healthy controls were shown individually. Comparison between the three groups was undertaken by one-way ANOVA, followed by Tukey’s multiple comparison test. ROC analysis of DNT cells and patients with post-transplant infections were performed using GraphPad Prism 8 (GraphPad Software, La Jolla, CA, USA). Correlation between DNT cells and CD4/CD8 ratio was performed using the Pearson correlation test. (INF: n = 9; NI: n = 10; HC: n = 12). * *p* < 0.05, ** *p* < 0.01.

We also found an expansion of DNT cells in pre-LTx patients who developed post-transplant infections, with a median proportion of 11.65% (IQR: 6.33%, 16.77%), while the numbers of DNT cells in pre-LTx patients without post-transplant infections and healthy controls (HCs) were 5.63% (IQR: 4.35%, 7.85%) and 4.99% (IQR: 3.79%, 6.33%), respectively (Figure 2A). The receiver operating characteristic (ROC) analysis showed that the threshold at which the area under the ROC curve (AUC) of DNT cells may be used to distinguish patients with post-transplant infections from patients without infections after LTx was 0.8353 (95% CI: 0.6591–1.000, *p* = 0.0113) (Figure 2B). The cut-off level of the pre-LTx DNT cell level was 11.35%. The sensitivity, specificity, and likelihood ratio (LR) were 60%, 90%, and 6.000, respectively. Similar to the CD4^+^ T cells, DNT cells were also heterogeneous with a composition of naïve (CD45RA^+^CD62L^+^), central memory (CM: CD45RA^−^CD62L^+^), effector memory (EM: CD45RA^−^CD62L^−^), and terminally differentiated effector subsets (EMRA: CD45RA^+^CD62L^−^) in both healthy donors and the two groups of LTx patients (Figure 2C). Although no significant difference between each T cell subset was found in the two groups of LTx patients, there were relatively fewer naïve DNT cells in pre-Tx patients who developed infections after LTx (Figure 2D–G). 

**Figure 3:**
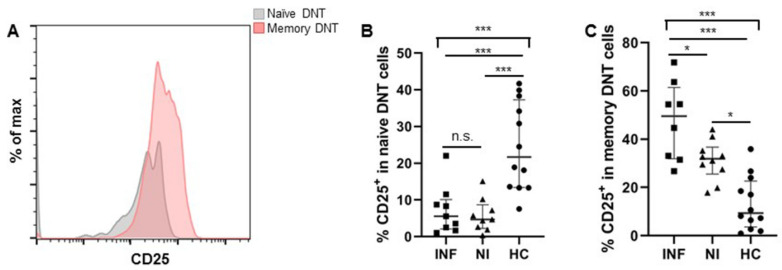
Memory DNT cells in pre-LTx patients expressed more CD25 in patients with post-transplant infections. (**A**) The expression level of CD25 on naïve and memory DNT cells was showed from a representative patient before LTx. (**B**,**C**) Expression of CD25 on naïve and memory DNT cells in the two groups of patients (INF & NI) and healthy controls were shown individually. (INF: n = 9; NI: n = 10; HC: n = 12). * *p* < 0.05, *** *p* < 0.001.

When we focused on the activation status of DNT cells in the two groups of LTx patients, memory DNT cells were more activated, with higher CD25 expression in patients who developed post-transplant infections, while the activation status of naïve DNT cells in the two patients’ groups were comparable regarding CD25 expression (Figure 3A–C).

#### 3.1.4. CD8^+^ T Cells Were Exhausted in Pre-LTx Patients Who Developed Post-Transplant Infections

**Figure 4:**
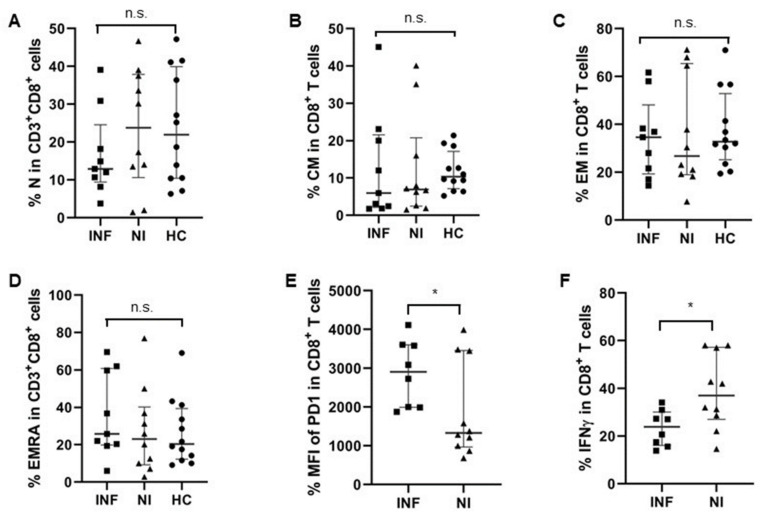
Pre-transplant CD8^+^ T cells were more exhausted in patients with infections after LTx. (**A**–**D**) Percentages of CD8^+^ T cell subsets were similar in the two groups of patients. N: CD45RA^+^CD62L^+^ naïve T cells; CM: CD45RA^−^CD62L^+^ central-memory T cells; EM: CD45RA^−^CD62L^−^ effector memory T cells; EMRA: CD45RA^+^CD62L^−^ terminally differentiated effector subsets. (**E**) Mean Fluorescent Intensity (MFI) of PD-1 in CD8^+^ T cells was significantly higher in patients who developed infections after LTx, compared to those who had no infections after LTx. (**F**) Levels of IFNγ producing CD8^+^ T cells in patients with post-transplant infections were lower than those in patients who had no infections after LTx. (INF: n = 9; NI: n = 10; HC: n = 12). * *p* < 0.05.

As CD8+ T cells play such an essential role in the elimination of intracellular infections and can provide long-term protective immunity, we analyzed CD8^+^ T cells’ composition and function with respect to their IFNγ secretion capacity in pre-LTx patients. There was no significant difference regarding the proportions of CD8^+^ T cells together with their subsets like the naïve, CM, EM, and EMRA cell levels for the two groups of LTx patients (Figure 1C and Figure 4A–D). However, the Mean Fluorescent Intensity (MFI) of PD-1 in CD8^+^ T cells was significantly higher in pre-LTx patients who developed post-transplant infections, compared to those who had no infections after LTx. This indicates that CD8^+^ T cells in pre-LTx patients who developed post-transplant infections were exhausted before LTx (Figure 4E). Consistent with this finding, CD8^+^ T cells from pre-LTx patients who developed post-transplant infections produced less IFNγ in response to additional stimulation with PMA/Ionomycin, compared to patients who had no infections after LTx (Figure 4F).

#### 3.1.5. Increased DNT Cells Were Still Observed in Patients after LTx

**Figure 5:**
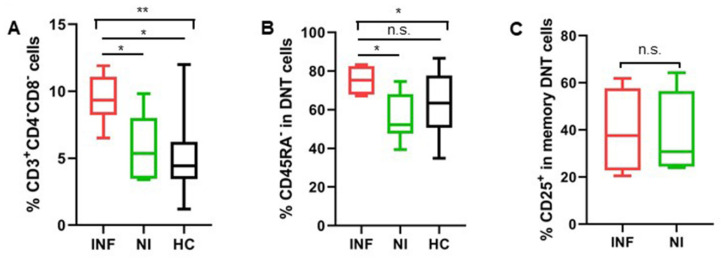
Increased DNT cell levels were still observed in patients who developed post-transplant infections after LTx. (**A**) Patients who developed post-transplant infections still had more DNT cells 2 weeks after LTx than patients without infections. (**B**) Patients with post-transplant infections had more memory DNT cells 2 weeks after LTx than patients without infections. (**C**) Expressions of activation marker CD25 were comparable in memory DNT cells for the two groups of patients. (INF: n = 9; NI: n = 10; HC: n = 12). * *p* < 0.05, ** *p* < 0.01.

We also performed the same assessments of T cells’ heterogeneity and function in transplant recipients after LTx. Interestingly, the expansion of DNT cells was still observed 2 weeks after LTx in patients who developed post-transplant infections, compared to those who had no infections after LTx (Figure 5A). Furthermore, these DNT cells also had more CD45RA^−^ memory cells than those DNT cells in patients without infections after LTx (Figure 5B). However, unlike the pre-LTx patients, there was no significant difference regarding the expression of CD25 on memory DNT cells in the two groups of patients after LTx (Figure 5C).

## 4. Discussion

Post-transplant infection, which mainly occurrs within the first month after transplantation, is one of the key issues for morbidity and mortality in transplant patients [7]. Biomarkers which can predict the development of infections after LTx are of great value for the optimization of immunosuppressive therapy and prevention of infections after LTx. Complementary systems, such as measuring serum complement component 3 levels at 2 weeks after transplantation, were reported as important for predicting 90-day mortality in living donor liver transplantation [36]. Mysore has also reported that patients with the highest expression of PD-1^+^Tim-3^+^ T cells in the memory compartment before transplantation had increased incidence of infections after liver transplantation, especially within the first 90 days [13]. We also observed in this study that CD8^+^ T cells in pre-transplant patients who developed post-transplant infections were more exhausted, with higher PD-1 expression and lower IFNγ production (Figure 4E,F). However, we didn’t find a significant correlation between PD-1 expression on CD8^+^ T cells and post-transplant infections. The combination of several co-inhibitory markers, such as Tim3, might be necessary to gate the double-positive population in memory CD8^+^ T cells.

The etiologies of the patients in this study is rather limited; therefore, we compared the infections of patients before LTx and found that 2/9 patients who developed post-transplant infections had a bacterial infection 1 month before LTx, but both were well-treated at the time of LTx. In the 10 patients who had no infection within 6 months after LTx, 1 had peritonitis but was well-treated 2 months before LTx. Former infection might play a role in the development of post-transplant infections, but, in our cohort, previous infections before LTx in both groups were comparable and well-treated. As the level of serum immunosuppressant may also play a role in post-transplant infections, we compared the serum CsA level in the two groups and found no significant difference (Table 1). For a more precise comparison of serum immunosuppressant and infections, large cohort studies are still needed.

DNT cells have been previously reported to be closely related to autoimmune/inflammatory conditions [37]. Their regulatory and pathogenic functions are still controversial [38]. Yang et al. performed a deep analysis of mouse DNT cells using single-cell RNA sequencing technology that showed the heterogeneity of both naïve and activated DNT cells [34]. However, similar data on human DNT cells are still very limited. In this study, we found that human DNT cells in pre-LTx patients before LTx were generally elevated compared to healthy controls, which may indicate their suppressive contribution to the patients’ compromised immune status. Secondly, the circulating DNT cells in patients before LTx were heterogeneous with the composition of naïve, central-memory, effect-memory, and terminally differentiated effector subsets, such as CD4^+^ and CD8^+^ T cells. However, circulating DNT cells in patients before LTx had fewer naïve and more memory subsets, especially effector-memory cells, compared to the healthy controls. Patients who developed infections after LTx had even more memory DNT cells, with more CD25 expression. As the α-chain subunit of the IL2 receptor, IL2 played an important role in T cells’ survival and proliferation by binding to the IL2 receptor. In DNT cells, IL2 could increase the resistance to apoptosis and enhance their suppressive capacity toward CD4^+^ T cells, thus prolonging the survival time of the allograft skin in mice [29,39]. Therefore, a higher expression of CD25 on DNT cells in pre-LTx patients who developed post-transplant infections may also contribute to the enhanced suppressive capacity in these patients. 

Furthermore, CD8^+^ T cells in patients before LTx who developed post-transplant infections were more exhausted, with higher PD-1 expression and less IFNγ secretion. Thus, the expanded and activated DNT cells in these patients may exert an immunosuppressive function and contribute to the compromised CD8^+^ T cells’ immunity in these patients [40,41]. Additionally, DNT cells could proliferate spontaneously in the steady state and protect mice from infections with a live vaccine strain of *Francisella tularensis* [37,42]. However, due to the limited volume of blood samples we could obtain from pre-LTx patients, further proliferation assays could not be performed with sorted DNT cells. However, the correlation and prediction values of DNT cells in these patients for post-transplant infections is important for optimized and individualized immunosuppressive treatment for patients after LTx. Thus, in addition to the classical CD4^+^ and CD8^+^ T cells, the proportion and activation of DNT cells should also be an important parameter in transplant patients for evaluating their immune status.

Our data showed that expanded DNT cells in pre-LTx patients correlated with post-transplant infections, especially within the first 6 months after LTx. This may help to identify patients at risk for post-transplant infections; therefore, immunosuppressive therapy for these patients should be carefully adjusted to account for potential infections after LTx. This personalized approach, by monitoring patients’ T cell status, is the future direction for coordinating the immunosuppressive therapy and management of patients after LTx.

This research also had limitations, such as a small sample size who had complete clinical visits, in the final analysis. Due to the limited of blood volume we could withdraw from the transplant patients, we were unable to obtain enough cells to perform fluorescence-activated cell sorting and a subsequent suppressive assay on the DNT cells. We also monitored the serum immunosuppressant level during the whole period. Although no significant difference was observed between the two groups of patients, with or without post-transplant infections, all patients involved in this study were, coincidentally, under Cyclosporine A treatment. However, Tacrolimus is also a standard immunosuppressant used in our transplant center. We had more patients under Cyclosporine A treatment in this study only because we had more supply of Cyclosporine A than Tacrolimus in the hospital during the project period. Hence, it remains unknown whether the type of calcineurin inhibitor used has any influence on post-transplant infections. Concerning the strong correlation between DNT cell levels in pre-LTx patients and post-transplant infections, in a future study, a large cohort of patients under different immunosuppressant treatments, and from multiple centers, should be involved, to further assess the impact of T cells status in pre-LTx patients on post-transplant outcomes.

## Figures and Tables

**Table 1:** Characteristics of patients before LTx and healthy controls.

Characteristics	Post-LTx Patients		HCs (n = 12)	*p*
	Infection after LTx (n = 9)	No Infection after LTx (n = 10)		
Age (years), mean ± SD	50.89 ± 10.89	44.90 ± 9.54	46.12 ± 10.92	0.33
**Gender, n**				0.78
Female	2	2	4	
Male	7	8	8	
Primary liver disease, n				0.94
Hepatitis B	5	9	NA	
Hepatitis C	2	1	NA	
HCC	4	5	NA	
MELD scores, median (IQR)	27.00 (26.00, 28.50)	27.00 (24.50, 31.50)	NA	0.97
Cyclosporine A, median (IQR)	525.0 (371.6, 617.1)	539.5 (328.7, 713.7)	NA	0.43

LTx: Liver transplantation; HCs: healthy controls; IQR: interquartile range; HCC: Hepatocellular carcinoma; MELD: Mayo End-Stage Liver Disease. The *p*-value of age and gender were generated from the two patient groups and the heathy control group using one-way ANOVA. The *p*-value regarding the primary liver disease, MELD scores, and concentration of Cyclosporine A postdose levels (C-2) were calculated from the two patient groups using the Mann–Whitney test.

**Table 2:** Details of patients who developed infections after LTx.

Patient No.	Age (Y)	Gender	MELD Scores Pre LTx	Time of Infections after LTx (D)	Symptoms	Organisms Infected	Treatment
1	61–65	M	27	32	Fever, cough,	Klebsiella	piperacillin-tazobactam
2	41–45	F	29	14	Leukopenia, nausea, fever	Fungal infection; Staphylococcus	Caspofungin Acetate meropenem
3	46–50	M	24	30	Nausea, vomiting, abscess	Staphylococcus	Piperacillin-Tazobactam
4	46–50	M	26	62	Respiratory distress, fevers, cough	Strep pneumoniae	piperacillin-tazobactam
5	31–35	M	28	10	Fever, abdominal pain, nausea	E.coli sepsis	piperacillin-tazobactam
6	61–65	M	27	92	Hypopiesia, diarrhea, nausea	Staphylococcus; Fungal infection	Meropenem Caspofungin Acetate
7	45–50	M	26	12, 58	Fever, vomiting	E.coli UTI in combination with fungal ascites infection	piperacillin-tazobactam Caspofungin Acetate
8	61–65	F	30	30, 122	Fever, cough, pneumonia	Pseudomonas aeruginosa, influenza B viruses	Cefoperazone Sodium and Sulbactam Sodium, Oseltamivir
9	56–60	M	27	51	Nausea, vomiting, abscess	Staphylococcus; Fungal infection	Piperacillin-Tazobactam Caspofungin Acetate

## Data Availability

The datasets analyzed during the current study are available from the corresponding author on reasonable request.
